# A high-precision wound healing assay based on photosensitized culture substrates

**DOI:** 10.1038/s41598-024-59564-9

**Published:** 2024-04-20

**Authors:** Saphia Azzam, Lea Tomasova, Carina Danner, Michael Skiba, Maren Klein, Zeno Guttenberg, Stefanie Michaelis, Joachim Wegener

**Affiliations:** 1 Institut Fuer Analytische Chemie, Chemo- & Biosensorik, Universitaet Regensburg, Universitaetsstr. 31, 93053 Regensburg, Germany; 2 Ibidi GmbH, Lochhamer Schlag 11, 82166 Graefelfing, Germany; 3 Fraunhofer-Institut Fuer Elektronische Mikrosysteme Und Festkoerper-Technologien EMFT, Universitaetsstr. 31, 93053 Regensburg, Germany

**Keywords:** Cell migration, Wound healing, Photosensitizer, Singlet oxygen, Reactive oxygen species, Phototoxicity, Metastasis, Biophysical methods, Cytological techniques, Imaging, Lab-on-a-chip, Microscopy, Biological techniques, Cell biology, Cell death, Cell migration

## Abstract

Quantitative assessment of cell migration in vitro is often required in fundamental and applied research from different biomedical areas including wound repair, tumor metastasis or developmental biology. A collection of assays has been established throughout the years like the most widely used *scratch assay* or the so-called *barrier assay*. It is the principle of these assays to introduce a lesion into an otherwise confluent monolayer in order to study the migration of cells from the periphery into this artificial wound and determine the migration rate from the time necessary for wound closure. A novel assay makes use of photosensitizers doped into a polystyrene matrix. A thin layer of this composite material is coated on the bottom of regular cell culture ware showing perfect biocompatibility. When adherent cells are grown on this coating, resonant excitation of the photosensitizer induces a very local generation of ^1^O_2_, which kills the cells residing at the site of illumination. Cells outside the site of illumination are not harmed. When excitation of the photosensitizer is conducted by microscopic illumination, high-precision wounding in any size and geometry is available even in microfluidic channels. Besides proof-of-concept experiments, this study gives further insight into the mechanism of photosensitizer-mediated cell wounding.

## Introduction

The locomotion of cells inside a multicellular organism is a phenotypical hallmark of several physiological or pathophysiological processes. Surveillance of the human body by immune cells and wound closure after injury are examples of the former, while the dissemination of aberrant tumor cells is an example of the latter^1^. Due to this overwhelming importance for health and disease, cell locomotion is also extensively studied in vitro to understand the underlying molecular mechanisms, to unravel the associated signal transduction and to find therapeutic interventions^2^. The term cell locomotion is, however, very general and includes different phenomena, in particular *migration* and *invasion*, which are rather different processes in experimental cell biology. Cell *migration* refers to the directed movement of cells along a 2D surface, like a basal membrane or a protein-coated plastic plate. Whether the cells migrate individually (single cell migration) or collectively (collective cell migration) in this assay depends on the cell type under study and the mechanical resilience of their cell–cell contacts. *Invasion* is defined as the cell movement through a 3D extracellular matrix (ECM), which requires adhesion, proteolysis of ECM components and eventually migration. Many different assays have been developed in the past to study cell *migration* or cell *invasion *in vitro using cell culture models of different complexity, ranging from immortalized cell lines to patient-derived in vitro models^2^. 

Since this study presents a novel approach to study 2D cell *migration* with unique advantages, the subsequent discussion is confined to this particular mode of cell locomotion. Most assays to study cell migration share that a well-defined opening in an otherwise complete cell monolayer is established either by wounding an intact cell layer (electrically, mechanically or optically) or by keeping cells out of a certain area of the growth surface by a removable barrier. After the barrier is removed, the cells are allowed to migrate in. The most popular and widespread assay belongs to the first group. It is based on growing the cells of interest to confluence before a lesion is introduced by mechanically scratching the cells away with a pipette tip or something similar. Accordingly, the assay is called *scratch assay*^3^. After wounding, the cells from the periphery of the lesion are allowed to migrate into the open space of the wound. Wound closure is monitored by time-lapse microscopy. The assay is easy to perform; it requires just a phase contrast microscope next to regular cell culture facilities. However, the wound size is difficult to control, in particular for cells with mechanically strong cell–cell adhesion as such cells may get removed collaterally, if they are in contact with the cells in the wounding path. This phenomenon critically affects the reproducibility of the assay. Moreover, the extracellular matrix in the wound path is completely undefined. It may have been removed or is still partially in place after the wound has been introduced. Since all cell debris has been cleared out of the wound path, the *scratch assay* does not mimic a physiological wound healing scenario realistically. The migrating cells don’t have to remove dead cells from the scratch by proteolysis before migrating in. Despite all these limitations, the assay is widely used but cannot be applied to cells in microfluidic channels with no access from the outside to create the initial lesion^4^. The so-called *barrier assay* also relies on an opening in an otherwise confluent cell layer. However, the opening is not introduced by scratching^5^. Instead, cell adhesion to a certain part of the growth substrate is prevented by the presence of a mechanical barrier that covers this particular area of the growth surface. Once the barrier is removed at a given time point of the experiment, the cells are allowed to migrate into the experimental lesion. The assay is also a *low-tech* assay not requiring any more than the barriers and a phase contrast microscope. It has much better reproducibility due to perfect control over wound size by the geometry of the mechanical barrier. However, there is also no cell debris in the area that is available for cell migration. The extracellular matrix in the wound is also hard to control, since it may form immediately after barrier removal by protein adsorption from the buffer or cell culture medium. Similar to the *scratch assay*, it cannot be applied to closed geometries like microfluidic channels, as barrier insertion and removal require access from the outside^6^. Recently, a technically different wound healing assay has been described that replaces mechanical wounding as performed in the *scratch assay* by electrical wounding^7^. Microscopic observation of wound healing is replaced by electrochemical impedance measurements. The latter approach is referred to as *electric cell-substrate impedance sensing*, or short ECIS, in the literature. The assay requires culture substrates with integrated thin film electrodes that the cells grow on. Once the cell layer is confluent, the cells residing on the electrode get efficiently killed by a lethal voltage or current pulse of 30 to 60 s duration. Only the cells on the electrode experience this electrical wounding, whereas cells in the periphery of the electrode remain unaffected. Thus, the wound size is perfectly controlled by the geometry of the electrode. After electrical wounding, the electrode is covered with cell debris. Cells migrating in from the periphery have to remove the dead cell bodies to open up space on the culture substrate to adhere to. In this respect, the assay produces a true wound healing scenario. The extracellular matrix on the electrode is not affected by the elevated electric field so that the native endogenous matrix composition produced by the cells, originally adhered to the electrode, is still in place. The electrical impedance of the cell-covered electrode is drastically different from a cell-free electrode since the intact cell bodies behave like insulating particles so that repopulation of the electrode by cell migration is assessable from time-resolved impedance readings without any microscopic observation. Since wounding and monitoring are completely software-controlled, this migration assay provides a high level of automation, a time resolution that is easily tuned to a few minutes and it is applicable to multi-well formats or microfluidic channels. The complete workflow of the impedance-based assay is performed in an incubator at physiological conditions. However, this approach requires extra equipment (impedance analyzer with integrated wounding module plus electrode arrays) that is typically not found in a standard cell culture lab but it is commercially available. Moreover, the wounding conditions are to some degree cell-type dependent and need to be optimized individually. Similar to the electric field-mediated cell wounding, a high-precision lesion may be introduced into a confluent cell layer by *laser ablation*^8^. Using lethal laser intensity in a microscopic setup allows creating wounded areas of any shape and size, leaving cell debris and extracellular matrix behind. Documentation of cell migration into the experimental lesion is again based on time-lapse video microscopy and subsequent image analysis. Due to the rather expensive hardware that is needed for cell ablation, the accompanying safety measures and the know-how for performing this assay, this technique is not very widespread and established. It is important to recognize the individual technical advantages and limitations of these different assays when comparing the results of different wound healing/migration studies. The constitution of the extracellular matrix in the wound and particular the presence of cell debris might affect the cells’ ability to migrate and close the wound. Moreover, it is noteworthy that wound healing may comprise to various degrees (i) cell migration and (ii) cell proliferation depending on the size of the wound, the cell type under study, the state of the cell cycle and the general metabolic situation of the cells^16^. All these experimental parameters need to be considered for a fair comparison.

This study describes a novel assay to study cell migration in vitro that introduces experimental lesions of any size and shape by illumination through a conventional fluorescence microscope with regular light sources. The low-intensity illumination is capable of killing cells in the light path, as the cells are grown on regular cell culture substrates that have been coated with a biocompatible functional layer containing a photosensitizer. Upon excitation, the photosensitizer molecules produce singlet oxygen ^1^O_2_ that kills the cells locally at the site of illumination but does not diffuse to neighboring cells due to its short lifetime. High-precision wounds are easy to obtain in cell layers grown on all types of culture substrates including multi-well formats and microfluidic channels using standard microscopic equipment available in most cell culture labs.

## Materials & methods

### Cell culture—general

All cell culture work was performed under a class II sterile bench (HERAsafe, Thermo Fisher Scientific Inc., USA). Non-sterile solutions as well as consumables were autoclaved for 20 min at 120 °C (DX-45, Systec, Germany) prior to use. Solutions and media were pre-warmed to 37 °C in a water bath (TW12, Julabo GmbH, Germany) before they got in contact with cells. The cells were routinely cultured at 37 °C and 5% (v/v) CO_2_ in a humidified incubator (Heraeus Function Line, Thermo Fisher Scientific Inc., USA). Cell culture media were changed every two to three days. Unless otherwise stated, all chemicals were purchased from Sigma-Aldrich GmbH (Germany). The cell lines were subcultivated once a week by means of standard trypsinization protocols using buffered trypsin solutions containing 0.05% (w/v) trypsin and 1 mM EDTA in PBSˉˉ. After cell detachment and spin down, the cell pellet was re-suspended in culture medium and split in a ratio of 1:20, based on the original cell density per cm^2^ of growth area. The cell count was determined using a Buerker hemacytometer (Marienfeld Superior, Germany).

### Cell culture—specific

*Normal Rat Kidney* (NRK; Leibniz Institute DSMZ GmbH, Germany) cells were cultured in Dulbecco’s Modified Eagle’s Medium (DMEM) containing 4.5 g/L D-glucose and 3.7 g/L NaHCO_3_ supplemented with 5% (v/v) fetal calf serum (Biochrom, Germany), 2 mM L-glutamine, 100 µg/mL penicillin and 100 µg/mL streptomycin. RAT1 *rat fibroblasts* were purchased from DSMZ (Germany) and cultured in high glucose DMEM as detailed above supplemented with 10% fetal calf serum (Gibco, Life Technologies, USA). *Human Umbilical Vein Endothelial Cells* (HUVEC; PromoCell, Germany) were cultured in *Endothelial Cell Basal Medium* supplemented with *Endothelial Cell Growth Medium Supplement Mix* (both from PromoCell). The cells were cultured until reaching 80–90% confluency before subculture. Routinely, cell culture substrates were not pre-coated with adhesive proteins except for HUVEC cells. HUVECs were grown in culture dishes coated with 0.45 µg/cm^2^ collagen IV (mouse, purchased from Corning, USA).

### Cell culture—live/dead staining

To visualize and document the wounded area with improved contrast, two similar fluorescence-based live/dead assays were conducted at various time points after wounding. (i) Invitrogen™ LIVE/DEAD viability assay: The assay kit contains ethidium homodimer-1 (EthD; λ_exc_ = 528 nm; λ_em_ = 617 nm) providing a red nuclear fluorescence in dead or injured cells. The stock solution of ethidium homodimer-1 in DMSO was diluted in PBS^++^ to a final concentration of 4 µM. The second fluorophore in this assay is calcein acetoxymethylester (CAM; λ_exc_ = 494 nm; λ_em_ = 517 nm) which is hydrolyzed to calcein in living cells providing a green fluorescent cytoplasm. The stock solution of CAM in DMSO was diluted in PBS^++^ to a final concentration of 2 µM. To stain the cells after optical wounding, the culture medium was removed; the cells were washed once with PBS^++^ and incubated with the staining solution containing EHD und CAM as detailed above in the dark for 1 h at 37 °C and 0% CO_2_. After incubation, the staining solution was removed, the specimen was washed with PBS^++^ and then imaged using an upright Nikon Eclipse 90i Confocal Laser Scanning Microscope (CLSM) in combination with a 10 × objective (NA = 0.25). (ii) In a second assay the cells were stained with a solution containing 8 µg/mL fluorescein diacetate (FDA; λ_exc_ = 498 nm; λ_em_ = 517 nm) and 20 µg/mL propidium iodide (PI; λ_exc_ = 535 nm; λ_em_ = 617 nm) in serum-free medium after the optical wounding. FDA and PI were both purchased from Sigma-Aldrich (USA). Similar to assay (i), red-emitting propidium iodide stains the nuclear DNA of dead cells. Fluorescein diacetate is converted to fluorescein in the cytoplasm of living cells so that living cells show a bright green cytoplasmic fluorescence. The samples were incubated with the staining solution for 10 min at 37 °C. Then, the monolayers were washed with serum-free medium. Phase contrast and fluorescent micrographs of the wounded areas were taken with the Nikon TiE Eclipse inverted microscope using a 10 × objective (CFI Plan Fluor DL Phase, Nikon) and a CCD camera ORCA-Flash 4.0-LT (Hamamatsu Photonics, Japan). All microphotographs were processed using NIH ImageJ.

### Cell culture—staining for apoptosis

Apoptosis in cells subjected to the wound healing assay was studied by the CaspaTag™ Caspase 3,7 Assay Kit (Sigma-Aldrich GmbH, Germany) which reports on mid-stage apoptosis. This assay monitors the activity of caspase-3 and caspase-7. The test principle is based on a covalent binding of fluorescein-labeled inhibitors of caspases-3 and -7 (FLICA; λ_exc_ = 488 nm; λ_em_ = 510 nm). The fluorescent caspase inhibitors were diluted in culture medium 1:30 (v/v), added to the cells and incubated at 37 °C and 5% CO_2_ for 1 h. After this initial incubation, the CaspaTag™ staining solution was replaced by serum-free medium containing propidium iodide (PI) for staining of dead cells (see above) for 5 min at 37 °C and 5% CO_2_. After washing the cells once with the washing buffer included in the kit, cell staining was documented in the washing buffer using an epifluorescence microscope.

### Preparation of photosensitizer-functionalized culture substrates

To prepare the photosensitized culture substrates, a solution of 10% (w/v) polystyrene (PS) and 3% (w/w relative to the mass of PS) platinum(II)-5,10,15,20-tetrakis-(2,3,4,5,6-pentafluoro-phenyl)-porphyrin (PtTFPP, Porphyrin Systems e. K., Germany) was prepared in toluene and stirred at 900 rpm overnight in the dark. A solution of 10% (w/v) PS in toluene was used to prepare non-functional control substrates. Round cover glasses (12 mm diameter; 130–160 µm thickness; Marienfeld Superior, Germany) as well as µ-dishes (35 mm diameter; ibidi GmbH, Germany) with a glass bottom were used as carrier materials to be coated with the functional polymer layers. Before use, the cover slips were washed in a 5% (v/v) Elma Clean 10 solution (Elma Schmidbauer GmbH, Germany) for 30 min at 70 °C in an ultrasonic bath. After rinsing the cover slips with distilled water, they were bathed twice in distilled water for 30 min at 70 °C in an ultrasonic bath and dried overnight at 37 °C. Before spin coating, the cover slips were additionally washed with acetone and isopropanol before drying for 30 min at 130 °C.

The functional layer was applied using a commercially available spin coater (WS-400BZ-6NPP/Lite, Laurell Technologies Corporation, USA). The procedure is sketched in Figure S1 (cf. Supporting Information). Before either of the polymer solutions (functional layer or control) were applied, the cover glasses and µ-dishes were spin-coated with 50 µL and 200 µL of the adhesion promoter TI Prime (MicroChemicals GmbH, Germany) for 20 s at 3000 rpm, respectively (Figure S1A). The carrier materials coated with the adhesion promoter were activated for 10 min at 130 °C (Figure S1B) and then spin-coated with 50 µL and 200 µL polymer solution at 2000 rpm for 1 min, respectively (Figure S1C). After evaporation of the solvent, the coated cover slips were immobilized in regular Petri dishes using a biocompatible silicone adhesive and dried for 24 h before use. The assay-ready substrates were stored in a dark and dry environment. Prior to any cell-based assay, the substrates were sterilized for 1 min in argon plasma (Plasma Cleaner PDC-002, Harrick Plasma, USA).

### Characterization of the functionalized substrates

*Film thickness*: The thickness of the functional layer as deposited on the glass cover slips by spin-coating was determined via optical profilometry (nanoAnalytics GmbH, Germany). For this, the functional layer was locally scraped off the glass substrate again so that the resulting height difference to the surface of the intact coating corresponds to the thickness of the functional layer. To create a chemically homogeneous surface, the substrates were subsequently coated with a 10 nm thin gold layer (sputter coater Bal-Tec SCD050, Capovani Brothers Inc., USA).

*Biocompatibility*: Biocompatibility of the functionalized substrates was assessed by time-resolved (i) cell adhesion and (ii) cell proliferation studies. (i) For cell adhesion, NRK cells were suspended in culture medium and inoculated upon glass substrates that were either coated with the photosensitizer-doped polymer layer or the polymer layer only (2.5⋅10^5^ cells/cm^2^). Phase contrast micrographs were recorded in predefined time intervals (1 h / 3 h / 6 h / 24 h) to document cell adhesion and cell spreading with time using an inverted phase contrast microscope (Nikon DIAPHOT, Nikon Instruments Europe, Netherlands) with a 10 × objective (Plan, 10x, NA = 0.25, Nikon Instruments Europe, Netherlands). Images were taken with a digital camera (Nikon D 5000, Nikon GmbH, Germany). (ii) Cell proliferation was assessed by seeding a suspension of NRK cells in culture medium (2.5⋅10^4^ cells/cm^2^) upon photosensitizer-doped surface coatings or a corresponding control. Phase contrast micrographs were taken 24 h, 48 h, 72 h and 96 h after cell seeding to document the degree of surface coverage using an inverted phase contrast microscope (Nikon DIAPHOT) with a 4 × objective (Plan, 4x, NA = 0.13, Nikon Instruments Europe, Netherlands). Culture medium was exchanged every two days in proliferation assays.

### Workflow of the wound healing assay

For the optical wounding experiments, the cells were suspended in cell culture medium and seeded on the functionalized coatings at a density of 2.5⋅10^5^ – 5.0⋅10^5^ cells/cm^2^. After 24 h of incubation (37 °C, 5% CO_2_, humidified atmosphere), the medium was exchanged for fresh cell culture medium. Optical wounding was conducted 48 h after cell seeding, when a confluent cell layer was established. The substrates with the confluent cell layers were placed on the stage of two different microscope setups to demonstrate the broad applicability of the new assay: (i) an *inverted wide-field* or (ii) an *upright confocal laser scanning* microscope.

(i) The *inverted wide-field* microscope Nikon TiE Eclipse (Nikon GmbH, Germany) was equipped with a high-intensity mercury arc lamp (Intensilamp, Nikon), a V-2A filter cube (excitation: BP 380–420 nm; barrier: LP 450 nm), and a 10 × objective (CFI Plan Fluor DL Phase). Filter and objective were both purchased from Nikon. The functionalized substrates were illuminated for 0.5–2 min using the V-2A cube with the focal plane adjusted to the cell layer. The experimental parameters of individual cell wounding are detailed for each experiment in the results section. The size of the wound was set manually by adjusting the fluorescence field diaphragm (Nikon) to yield the illumination field of 500–1000 µm in diameter, which corresponds approximately to the later wound size. After the optical wounding, the cell culture medium was replaced by fresh medium and the substrates were incubated for 30 min at 37 °C. Afterwards, the wounded cell layers were either fluorescently labeled with a live/dead stain to document wound size and geometry or the wounds were studied in regular intervals by phase contrast video microscopy to monitor the wound healing process.

(ii) Wounding was also conducted using the *upright confocal laser scanning* microscope Nikon Eclipse 90i (Nikon Instruments Europe, Germany) capable of exciting the samples with three different lasers: (a) λ_exc,1_ = 408 nm, P_408nm_ < 500 mW, barrier_1_: BP450-485 nm; (b) λ_exc,2_ = 488 nm, P_488nm_ < 50 mW, barrier_2_: BP515-545 nm; (c) λ_exc,3_ = 543 nm, P_543nm_ < 5 mW, barrier_3_: LP650 nm. Laser intensity was tailored to experimental needs by insertion of neutral density filters ND4 and ND8, reducing the laser intensity to 25% or 12.5%, respectively. For photochemical wounding, samples were excited at a wavelength of λ = 408 nm for an exposure time of 0.5 min, 1 min or 2 min with or without neutral density filters as specified for individual experiments. The laser light was focused to the sample plane by a 10 × dry objective (Plan, 10x, NA = 0.25, Nikon Instruments Europe) and moved across the sample in *line scan* mode, i.e. the point of illumination moves unidirectional along a preset linear wound path. After wounding, the medium (2 mL) was exchanged by fresh medium to remove detached, dead cells. The wound was documented by means of phase contrast microscopy 1 h after injury. For documentation, the inverted phase contrast microscope Nikon DIAPHOT with a 4 × phase contrast objective was used. The pictures were taken with a built-in digital camera.

### Perfusion experiment

For the wound healing experiments under flow, the coated substrates were fixed to a bottomless channel slide using its self-adhesive underside (sticky-Slide Luer I^0.8^; ibidi GmbH). HUVEC cells were suspended in cell culture medium yielding a cell density of 1.5 × 10^6^ cells/mL. A total of 200 µL of this cell suspension was seeded into the channel and incubated overnight at 37 °C. After cell attachment, the slide was connected to a perfusion set (yellow-and-green, inner diameter 1.6 mm, ibidi GmbH) filled with 12 mL of cell culture medium supplemented with antibiotics and connected to the ibidi pump system. The cells were cultured for 24 h experiencing a laminar flow of 1 dyne/cm^2^. Afterwards, the optical wounding was performed inside the channel using best practice parameters. After wounding, the laminar medium flow was increased to yield a shear stress of 7 dynes/cm^2^. Cell migration into the wounded area under these flow conditions was studied for 24 h by video microscopy with a time-lapse interval of 10 min.

### Video microscopy

Cell migration into the wounded areas was documented by phase contrast time-lapse microscopy, using the Nikon TiE Eclipse inverted microscope equipped with a motorized stage (TI-SH-W, Nikon), a 10 × objective (CFI Plan Fluor DL Phase, Nikon), a CCD camera ORCA-Flash 4.0-LT (Hamamatsu Photonics, Japan) and a stage top incubation system (ibidi GmbH, Germany). The device ensures stable environmental conditions for the duration of the experiment (37 °C, 5% CO_2_, 80% humidity). Image acquisition was started 30 min after the optical wounding and the wound healing was recorded for 24 h with a time-lapse interval of 10 min. The microscope was controlled with the *Micro-Manager* software. For quantification of the wound healing rate, the wounded area in selected microphotographs was analyzed with the ImageJ software (see below) in 2 h intervals starting 1.5 h after wounding.

### Quantification of wound size and wound healing rate

To determine the wound size as a function of time, phase contrast images were evaluated using the image processing program ImageJ (Wayne Rasband, NIH). A line was drawn by hand along the wound boundaries, which was followed by an automated calculation of the (encircled) wound area. The area obtained was converted (from pixel^2^ into µm^2^) using a phase contrast microscopic image of a Buerker chamber (Marienfeld Superior, Germany) for calibration. The results of the wounded area at individual time points were further analyzed to quantify the wound healing rate using OriginLab (Version 2020, OriginLab Corporation, USA).

### Human and animal rights

There were no animals involved in our study submitted to Scientific Reports. It is an entirely in vitro approach using cultured cell lines exclusively.

## Results & discussion

**Figure 1:**
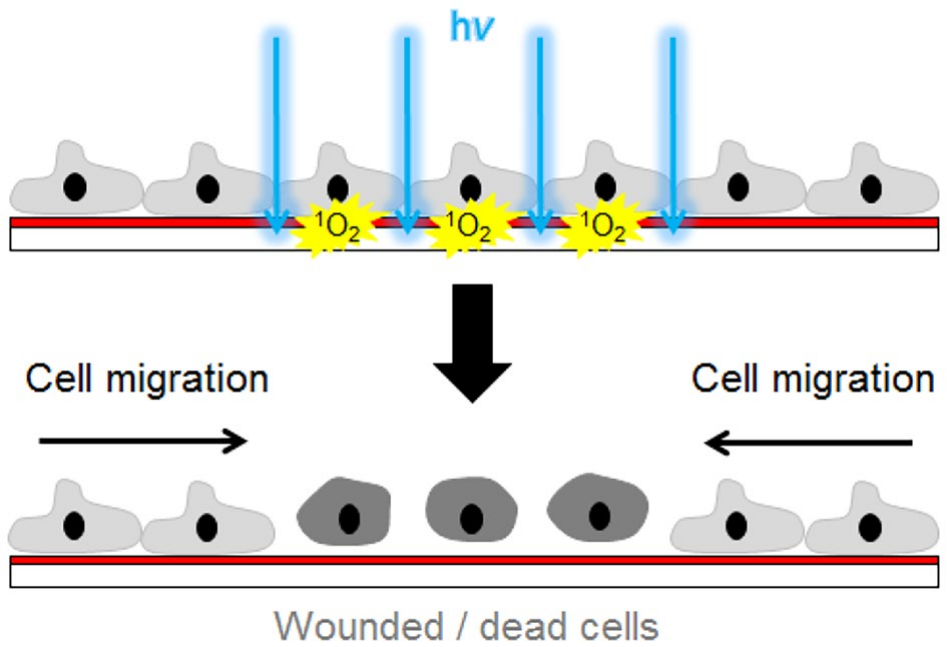
Principle of the optical wound healing assay: Adherent cells are grown on specialized cell culture dishes that are coated with a thin polystyrene layer which is doped with the photosensitizer PtTFFP. Upon illumination, the photosensitizer turns ^3^O_2_ into short-lived ^1^O_2_ which kills those cells that are residing on the functional layer at the site of illumination. After the lesion is introduced, vital cells from the periphery of the wound migrate into the center of the lesion and eventually close the wound. The assay provides wounds with microscopic precision and in any geometry or pattern.

The wound healing/migration assay as presented here is based on cell culture substrates that have been coated with a thin polystyrene layer doped with the photosensitizer platin(II)-5,10,15,20-tetrakis-(2,3,4,5,6-pentafluorophenyl)-porphyrin, or short PtTFPP. When PtTFPP is excited at a wavelength close to its absorbance maximum (λ_max_ = 395 nm), it turns ^3^O_2_ from the ambient air into toxic but short-lived ^1^O_2_ by means of photoluminescence collision quenching. Since PtTFPP was embedded in an oxygen-permeable matrix polymer (polystyrene), ^3^O_2_ is readily available for conversion. Due to its short life-time of just several µsec in aqueous solutions, ^1^O_2_ diffuses only very short distances before it relaxes back to ^3^O_2_^9–11^_._ This limited diffusive mobility of ^1^O_2_ enables a highly localized cell killing or wounding^12^. The toxic ^1^O_2_ is only capable of harming those cells that are residing on the functional coating at the site of ^1^O_2_ generation so that the wound size is precisely controlled by the geometry of the illumination spot. After wounding, the wounded cell bodies are left behind so that the subsequent healing corresponds in this respect to the natural wound healing process in animals. Figure 1 sketches the principle of the optical wound healing assay.

### Characterization of the photosensitive coating

Since the photosensitive coating is the core component of the new assay, it has been characterized in detail regarding its physical and biological properties. After coating glass substrates with the functional polymer as detailed in Material & Methods, the layer had an average thickness of (1.6 ± 0.1) µm as determined by optical profilometry. The thickness of the functional coating is controlled by the parameters of the spin coating process and the viscosity of the polymer solution. Even though we did not study the impact of film thickness on the wounding process in detail, the pool of our data suggests that there is no advantage in using thicker functional layers. This is in line with the rather short life-time of ^1^O_2_, which would relax back to ^3^O_2_ before reaching the cell-covered surface if it were produced deep inside a thick polymer layer. Thus, from the point of view of ^1^O_2_ production close enough to the surface, a significantly thinner functional layer would most likely provide similar results. But layer thickness is a trade-off between the sufficient ^1^O_2_ production and the coating process itself. With the µm thick layer, we avoided problems of inhomogeneous wetting during spin coating and the layers were stable and uniform for several months in air without any blebbing or peeling off.

Figure S2 (cf. Supporting Information) shows the absorbance spectrum of the photosensitizer PtTFFP in a polystyrene matrix (3% w/w) as it was used during development of this assays. The maximum absorbance is assigned to the Soret band at 395 nm. Two less intense absorbance bands are present in the green spectral band at 508 nm and 541 nm. For the wounding of cells, we used an excitation wavelength of 408 nm which is slightly red-shifted from the Soret band but still sufficiently intense to induce photo-luminescence that is quenched by ^3^O_2_ generating ^1^O_2_. It has been described before that molecules like PtTFFP that undergo intersystem crossing after excitation adopting a triplet state, are particularly well suited to produce ^1^O_2_^13,14^. It is noteworthy that cells residing on the functional layer will get also harmed upon prolonged exposure to white light of moderate intensity as used for bright field or phase contrast microscopy. However, unintended cell killing requires continuous exposure for several hours and is not an issue if the light source is dimmed down during video microscopy documentation. If continuous observation of the cells after wounding is needed with high light intensity, we recommend using an edge filter with an edge wavelength above the absorbance wavelength of the sensitizer during cell monitoring. This filter will avoid any unintended cell damage during long-term observation of cells adhered to the functional layer.

Regular cell culture ware is most often made from polystyrene—just as the polymer matrix of the functional coating used for optical wounding. Polystyrene surfaces are typically hydrophobic by nature and need to get activated (for instance by UV exposure) before they are suitable for cell and tissue culture^15^. Very similar properties have been observed for the functional coating in this project. After depositing the doped polymer layer, the surface was rather hydrophobic and poorly wettable. Thus, the functionalized substrates were exposed to argon plasma for 1 min to sterilize the device and make the surface more wettable which is a prerequisite for protein adsorption and subsequent cell adhesion.

**Figure 2:**
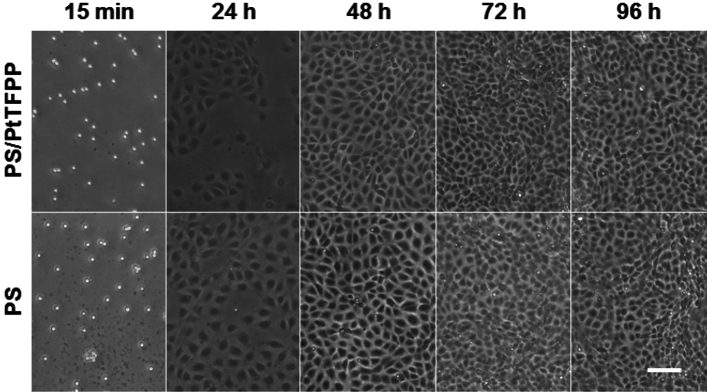
Proliferation of initially suspended NRK cells on a glass substrate coated either with a layer of photosensitizer-doped polystyrene (upper panel) or pure polystyrene (lower panel). Phase contrast images were taken 15 min, 24 h, 48 h, 72 h and 96 h after cell seeding. The cells were seeded in culture medium (DMEM high glucose) with a density of 2.5⋅10^5^ c/cm^2^. Scale bar: 100 µm.

In order to verify the compatibility of the functional layer with respect to cell adhesion and proliferation, we compared the time courses of cell adhesion and proliferation for initially suspended cells on PtTFPP-doped polymer layers (PS/PtTFFP) with the pure polystyrene (PS) coating. Figure 2 summarizes these experiments using Normal Rat Kidney (NRK) cells. Further tests using other cell lines confirmed these results. In all cases, culture substrates coated with the functional layer (PS/PtTFPP) did not show any significant difference in cell compatibility compared to a pure polystyrene (PS) coating. This applies to cell density at either time point just as much as to cell morphology. We haven’t observed any sign of cell culture incompatibility throughout our studies as long as the cell-coated substrates were not continuously exposed to light for several hours as indicated in the preceding paragraph.

### Proof-of-concept experiments

**Figure 3:**
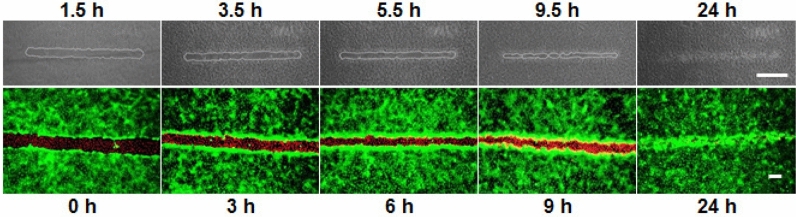
*Upper panel:* Wound healing/migration assay conducted with NRK cells grown to confluence on functionalized polymer coatings after optical wounding with a laser of λ = 408 nm using an upright CLSM. The substrates were illuminated for 1 min. An ND4 filter adjusted the laser intensity. Phase contrast micrographs were recorded 1.5 h, 3.5 h, 5.5 h, 9.5 h and 24 h after wounding. The wound edges were marked with the help of the image analysis software ImageJ. Scale bar: 400 µm. *Lower panel:* Wound healing/migration assay as in the upper panel. However, cells were studied by fluorescence microscopy after a vital stain using calcein AM (living cells, green)/EthD-1 (dead cells, red). Samples were stained 0 h, 3 h, 6 h, 9 h or 24 h after wounding. Scale bar: 100 µm.

Before discussing individual aspects of the optical wound healing assay, this paragraph provides proof-of-concept using the best practice parameters that evolved during assay development. Figure 3 shows the outcome of a typical assay in which NRK cells were grown to confluence on a culture substrate coated with the doped polymer layer as described above. Using an upright confocal laser scanning microscope, a straight wound has been introduced into the cell layer at λ = 408 nm excitation. The upper panel shows phase contrast micrographs of the wound starting 1.5 h after wounding (left) until the wound was closed by cell migration from the periphery 24 h after wounding (right). Since phase contrast micrographs provide only a rather limited contrast difference between living and dead cells, the wound edge has been labeled manually by a white line. The wound area is maximal 1.5 h after wounding and decreases continuously as time progresses. After 24 h the wound is completely closed by collective cell migration. As in any wound healing/migration assay, we cannot exclude that cell proliferation also contributes to wound closure^16^. The live/dead contrast as observed in phase contrast micrographs develops within the first 1–2 h after illumination. We take this as a strong indicator that the ^1^O_2_ generated by the photosensitizer does not kill the cells directly and immediately as, for instance, during cell lysis. The somewhat delayed cell death as judged from phase contrast micrographs is more likely the endpoint of a signaling cascade that was triggered by the accumulation of reactive oxygen species (ROS) inside the cytoplasm.

The lower panel in Fig. 3 illustrates a wounding experiment performed with confluent NRK cells under the same conditions as in the upper panel except for the readout. Instead of recording phase contrast micrographs, we stained the cell specimen with a set of two vital stains: calcein AM and ethidium homodimer. Whereas the former labels living cells with a green fluorescent cytoplasm, the latter introduces red fluorescence into the nuclei of dead cells. The fluorescent micrograph that was recorded immediately after wounding (0 h) provides a very clear live/dead contrast indicating the precision of the wound line. Please note that magnification is different for phase contrast compared to fluorescence contrast. The size of the wound decreases with time due to cell migration from the periphery. After 24 h the lesion is closed, as was also observed by phase contrast microscopy. The viable cells residing at the wound edge show a significantly brighter green fluorescence compared to cells farther away from the lesion. We interpret this highly reproducible observation by an increased metabolic activity of the cells at the wound edge when they are switching from a resting to a migratory phenotype. As the calcein staining requires enzyme activity to liberate the fluorochrome *calcein* from the fluorogen *calcein AM*, it seems plausible that the conversion rate is higher in cells with higher metabolic turnover. Similar observations have been made in other wound healing assays, for instance, after electrical wounding when the cells were stained by calcein AM^7^. We also noticed frequently some alignment of the cells towards the center of the wound that was differently expressed dependent on cell type.

**Figure 4:**
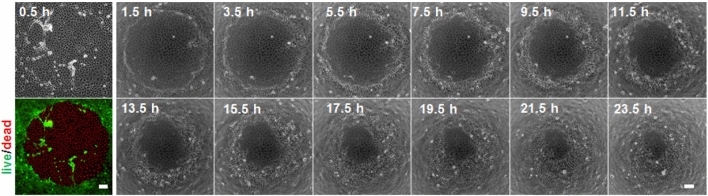
Optical wounding using a wide-field fluorescence microscope. The phase contrast micrograph (first column, upper panel) and fluorescence micrograph (first column, lower panel) show the wound in a confluent monolayer of RAT1 cells 30 min after the optical wounding. Living cells are labeled green (FDA), whereas dead cells show red nuclei (PI). The wound was created by a two-minute illumination using a V-2A excitation filter (λ = 408 nm). The series of phase contrast micrographs provides a time-lapse recording of cell migration on the functionalized substrate starting 1.5 h after wounding using a 2 h interval. The wound area was analyzed with the image analysis software *ImageJ* to calculate the migration rate (cf. Figure 5). Scale bars = 100 µm.

The question arose whether a laser is required to excite the photosensitizer or whether light sources as used in regular wide-field fluorescence microscopes are also suitable for optical wounding. Figure 4 summarizes an optical wounding experiment conducted on the stage of such a wide-field fluorescence microscope. Confluent monolayers of RAT1 cells were used as model system. Instead of introducing a linear wound by means of a focused laser beam, we applied the excitation light through a circular field diaphragm. Micrographs on the very left show the situation 0.5 h after wounding in phase contrast or fluorescence contrast after live/dead staining. The remaining phase contrast micrographs document the progress of wound healing of one and the same wound imaged at different times after illumination as indicated.

The initially recorded micrographs in phase and fluorescent contrast confirm successful cell killing with a circular geometry. All cells in the field of view during photosensitizer excitation have been killed whereas cells in the periphery are still vital. This experiment provides evidence for two different conclusions that are further supported by other experiments described below: (i) Light sources (e.g. mercury lamps) as used in wide-field microscopy are sufficiently powerful to achieve optical wounding. Compared to the use of a laser for wounding, light intensity was obviously lower using the mercury lamp so that successful wounding required two minutes of illumination instead of just one minute using the laser. As discussed later, optical wounding is dependent on the illumination light intensity. (ii) The optical wounding works for cells that originate from very different tissues and show correspondingly different phenotypes. NRK cells are tightly adhering to the substrate, grow in cobblestone morphology and express epithelial-like intercellular adherence junctions. RAT1 cells are fibroblasts and—as such—do not show strong adhesion to the extracellular matrix, and they do not express mechanically resilient adherence junctions. The independence of optical wounding of cell phenotypes is not surprising as the ^1^O_2_ is a rather generic stressor that produces other reactive oxygen species and oxidative damage in either cell type. We also applied the optical wound healing assay to (i) MDCK cells (canine epithelial cells); (ii) HaCaT cells (human keratinocytes); (iii) L929 cells (murine fibroblasts); (iv) MCF-7 cells (human breast cancer cell line); (v) HT1080 cells (human fibrosarcoma); (vi) RCC cells (human kidney cancer cell line). The cell lines have been selected since they are frequently studied in wound healing / migration assays and show a broad variety of different in vitro phenotypes. The optical assay worked reliably for all these cell lines after some individual adjustment of the experimental parameters, most notably light intensity and illumination time.

**Figure 5:**
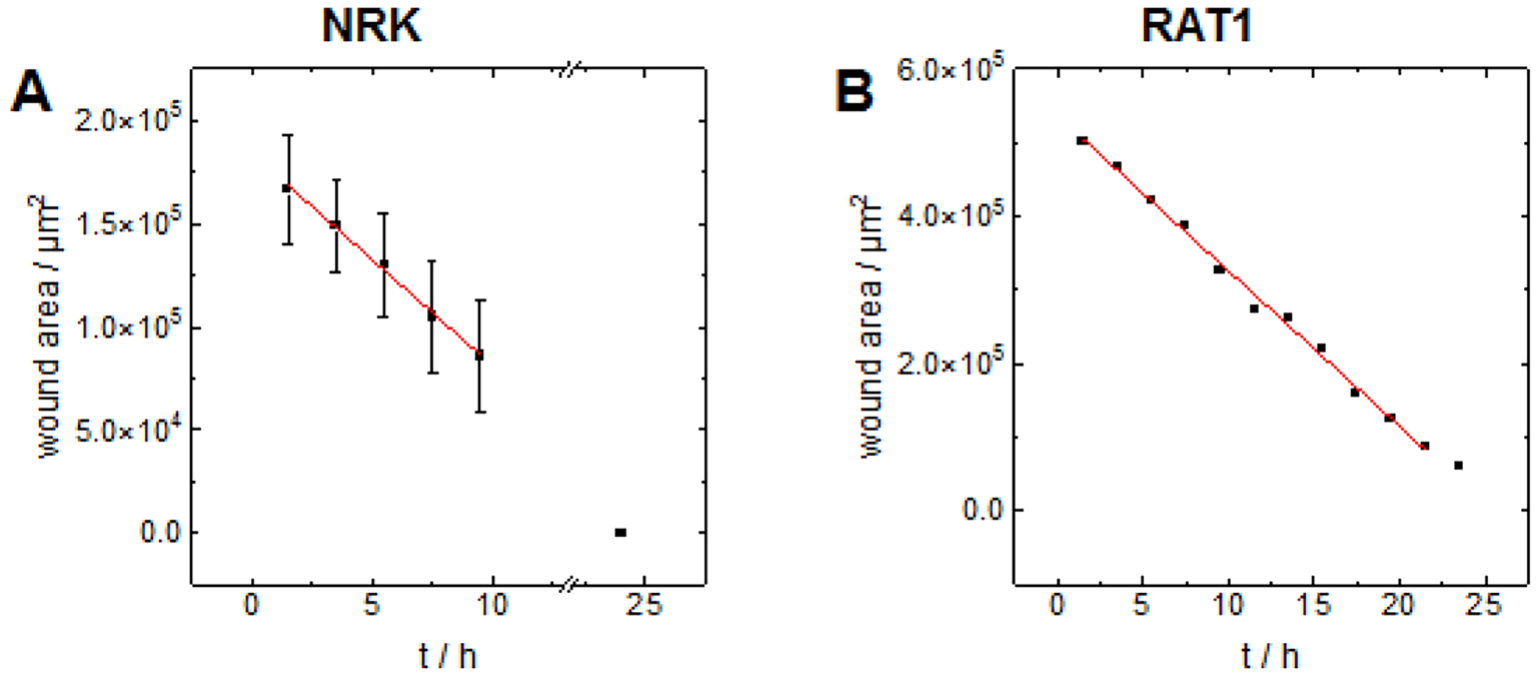
Measured wound area for NRK cells (A) and RAT1 cells (B) on functionalized glass substrates over time. The substrates were either illuminated for 1 min using a CLSM (A) or 2 min using an epifluorescence microscope (B) with a wavelength of λ = 408 nm and a 10 × objective. In case of RAT1 cells the illuminated area was manually adjusted by a diaphragm. In case of the NRK cells an ND4 filter was inserted to reduce the intensity of the laser light. The red line shows the linear regression of the data between the first and the fore last time point. From the negative slope of the linear regression, migration rates can be determined. For RAT1 cells the migration rate is (21.1 ± 0.5)⋅10^3^ µm^2^/h, for NRK cells it is (10.3 ± 0.4) ⋅10^3^ µm^2^/h. (A) Data depicted for NRK cells: mean ± SD, n = 5.

Conducting wound healing assays to quantify cell migration does not only require efficient killing but also documenting the wound closure process. In this respect, the optical wound healing assay described here is not different from other assays using mechanical wounding or barriers. However, compared to those assays in which the lesion is cell-free either because the cells have been scraped off or they did not have access to the area of the substrate initially, the wounded area is covered by dead cells. On the one hand, the presence of dead cells in the wound corresponds more closely to a real wound and enables more realistic wound healing/ migration experiments. On the other hand, it makes segregation of the wounded versus not-wounded area more difficult in phase contrast micrographs. But as visualized in Fig. 3, the image analysis platform *ImageJ* allowed marking the wound edges rather accurately and determining the wound area as detailed in Materials & Methods. Figure 5 summarizes the outcome of such a quantitative analysis for experiments performed with NRK cells using a confocal laser scanning microscope (A) next to the results of experiments recorded for RAT1 fibroblasts using a wide-field microscope for wounding (B). Whereas (A) provides the average and standard deviation of five individual experiments as mean ± standard deviation to illustrate the scattering that goes back to differences in substrate preparation, cell culture and cell age, (B) reports on a single experiment (cf. Figure 4). In either case, the graph shows the time course of the remaining wounded area as a function of time after illumination.

For a linear wound with a high aspect ratio (cf. Figure 3), the wound area decreases approximately linearly with time as long as the cells migrate with a constant migration velocity. In this particular case only migration starting from the long side contributes significantly to wound closure. The data in Fig. 5A confirm a linear dependence on time. The slope of the linear regression line amounts to (10.3 ± 0.4)⋅10^3^ µm^2^/h. The wound length (long side) was estimated to be 1500 µm from the micrographs in Fig. 3 so that the areal migration rate (given in µm^2^/h) translates into a linear migration rate of (6.9 ± 0.5) µm/h for NRK cells. Migration into a circular wound (cf. Figure 4) is more complicated to model as the wound area depends on the square of the wound radius r^2^. Strictly speaking, linear approximation is only valid for moderate wound healing progression. However, the data in Fig. 5B indicates a linear behavior even for the first 20 h of the experiment so that calculating the linear migration rate for the initial wound size at time zero is well justified. The initial perimeter of the circular wound at time zero amounts to 2350 µm which translates the areal migration rate of (21.1 ± 0.5) ⋅10^3^ µm^2^/h, as determined from the linear regression, into a linear migration rate of (9.0 ± 0.3) µm/h for RAT1 cells.

To validate the new assay, we performed benchmarking experiments as detailed in the supporting information (cf. Figure S5 and accompanying explanation). In these experiments we used the barrier assay (ibidi inserts) as a reference. Experimental details are given in the supporting information. In short, L929 mouse fibroblasts were studied in the optical wound healing / migration assay in the absence and presence of 10% serum in the culture medium. As expected, the areal wound healing rate was higher in presence than in absence of serum in the medium. The assay returned a 2.7 times higher areal wound healing rate in the presence of serum compared to its absence. The barrier assay was conducted under exactly the same conditions. For L929 cells we found that the areal migration rate to be 1.8 times higher in presence of serum compared to its absence. So both assays report correctly on the impact of serum on cell migration even though they showed individual relative increases. Since we used cells of the same passage number in those two assays, we assign the observed differences to the presence of cell debris and extracellular matrix in the optical assay but not in the barrier assay. The same argument applies to the significantly different wound healing rates under both conditions returned by the two assays. The presence of cell debris and an intact ECM in the wounded area provides an experimentally different situation that leads to individual readouts.

### Details of the wounding process: is resonant excitation necessary?

**Figure 6:**
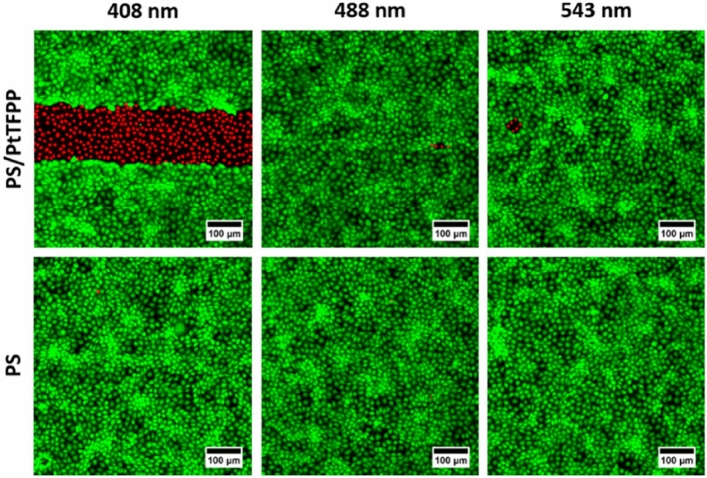
Fluorescence micrographs (CLSM) of confluent NRK cells grown on glass slides coated with PS/PtTFPP (upper panel) or PS (lower panel) that were illuminated for 2 min with 408 nm, 488 nm or 543 nm laser light. The cells were stained by a calcein AM / ethidium homodimer vital stain as detailed in Materials & Methods. Significant wounding only occurs when the growth substrates are coated with PS/PtTFPP functional layer excited at the resonant wavelength of the Soret band at 408 nm. Illumination of control substrates (PS) does not introduce wounds into the cell layer at any of the exposure conditions. The scale bar corresponds to 100 µm.

The optical wounding relies on exciting the photosensitizer PtTFFP at the wavelength of maximal absorbance which is the Soret band at approximately 400 nm. To prove that cell killing is not unspecific and independent of the photosensitizer, we conducted control wounding assays. In a first assay, the functionalized substrates were exposed to two other wavelengths that are often used in cell-based assays: 488 nm (FITC, GFP) and 543 nm (TRITC). The upper panel of Fig. 6 compares the appearance of the cell layer after exposure to 408 nm, 488 nm and 543 nm by means of fluorescence micrographs recorded after the calcein AM/ethidium homodimer vital staining.

The experimental conditions were identical except for the different lasers used for excitation. The illumination time was set to two minutes in all three exposure scenarios. The fluorescence micrographs in Fig. 6 (upper panel) reveal a significant wounding only for those cells that were exposed to light of 408 nm. However, the lasers that were used in these experiments had a rather different output power. The 408 nm laser provided approximately 500 mW, while the 488 nm laser has a nominal power of just 50 mW. The output power of the 543 nm laser was just in the order of 5 mW. Therefore, a second set of control assays was conducted that used polystyrene coatings without the photosensitizer (Fig. 6, lower panel). After vital staining, neither of the samples revealed any indication of cell wounding at any of the wavelengths applied for the same exposure as used in the upper panel. These experiments demonstrate that the microscopic wounding is indeed based on the presence of the photosensitizer PtTFFP and its excitation at its resonant wavelength (Soret band).

### Details of the wounding process: the impact of ^1^O_2_ scavengers

**Figure 7:**
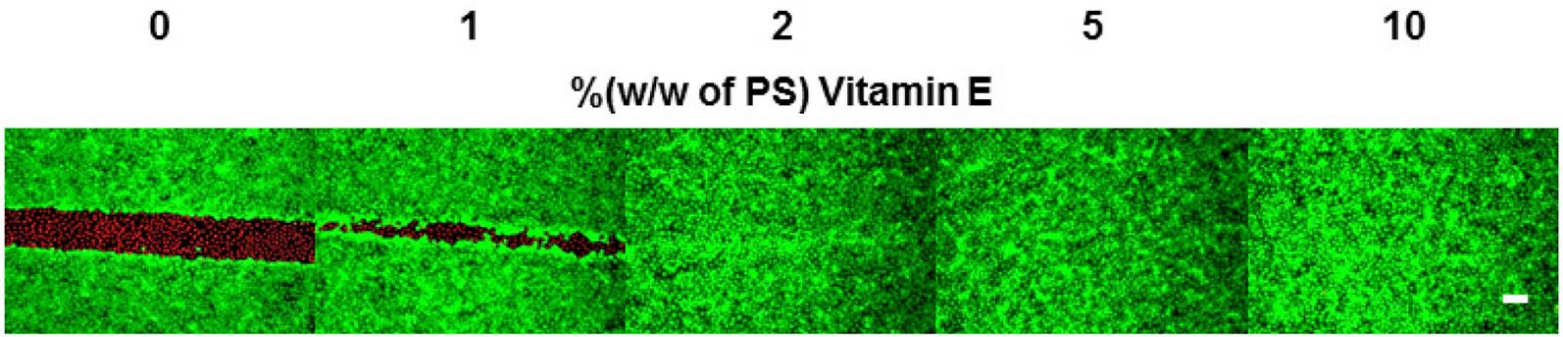
CaAM (green)/EthD-1 (red) assay of NRK cells on functionalized glass substrates containing different vitamin E concentrations after optical wounding with a laser of λ = 408 nm at the upright CLSM. Vitamin E concentrations of 0%, 1%, 2%, 5% and 10% (w/w of PS) were used. The illumination time was set to 1 min. An ND4 filter was inserted. Scale bar: 100 µm.

Significant optical cell killing requires the presence of the photosensitizer and its resonant excitation. To demonstrate the mediating role of ^1^O_2_, we prepared functional coatings that were additionally supplemented with an established ^1^O_2_ scavenger: α-tocopherol, a member of the vitamin E family^17^. Since α-tocopherol is hydrophobic and poorly soluble in water, it readily dissolves in the polystyrene / PtTFPP / toluene solution and was, thus, deposited inside the functional layer responsible for wounding. Different coatings were prepared with increasing content of α-tocopherol starting from 1% (w/w relative to the mass of polystyrene) to 10% (w/w). Figure 7 shows the outcome of wounding assays with the differently doped functional layers when confluent NRK cells were exposed to a one-minute illumination with the 408 nm laser. Whereas in the absence of α-tocopherol, the typical wound was generated by 408 nm light exposure, the wound size decreases significantly when the ^1^O_2_ scavenger is present in just 1% (w/w). Higher concentrations completely block the wound formation.

α-Tocopherol is known to quench ^1^O_2_ by physical (relaxation back to ^3^O_2_) or chemical mechanisms (oxidation of the scavenger) ^18^. Therefore, this experiment strongly suggests that ^1^O_2_ formation is responsible for the observed optical killing of the adherent cells. It is noteworthy that a single α-tocopherol molecule has the capacity to detoxify 40 to 120 molecules of ^1^O_2_ by physical quenching before its scavenging capacity is lost^19^.

### Details of the wounding process: do the wounded cells undergo apoptosis?

**Figure 8:**
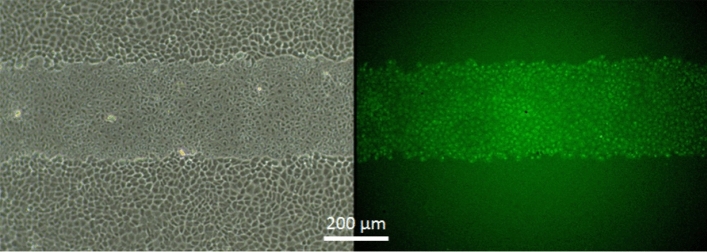
Confluent NRK cell layers after optical wounding for 2 min using the photosensitive functional coating of standard composition. (**A**) Phase contrast micrograph with a clearly visible wound running horizontally. (**B**) Fluorescent micrograph of the same field of view, after the cells have been treated with a fluorescently labeled inhibitor of caspases 3 and 7. The green cytoplasmic fluorescence indicates apoptosis of all cells in the wound but no sign of apoptosis in the periphery of the wound. Scale bar: 200 µm.

To get a better understanding of the mechanism of cell death induced by the photosensitizer-mediated generation of ^1^O_2_, we cultured NRK cells to confluence on the functionalized coating in standard composition. The cell layers were wounded for 2 min using a wide-field fluorescence microscope. After light exposure, the cells were allowed to rest for one hour before they were stained for apoptosis as described in Materials & Methods. For the detection of apoptosis, we made use of fluorescence-labeled inhibitors of caspases 3 and 7 (FLICA) ^20^. The fluorescent labels are membrane-permeable as long as they have not irreversibly bound to any of the two caspases. Accordingly, apoptotic cells show a brightly fluorescent cytoplasm when caspase 3, caspase 7 or both are expressed as a response to the onset of apoptosis. Figure 8 illustrates a typical staining that was observed with this assay after regular optical cell wounding using a two-minute illumination. The wounded cell area is clearly visible in phase contrast (left). The FLICA assay showed all cells in the wounded area labeled by green fluorescence. Accordingly, all cells expressed caspase 3, caspase 7 or both within one hour after wounding. As anticipated for the conditions applied here, the light-induced cell killing is indeed mediated by triggering the apoptosis cascades within the cells under study.

### Details of the wounding process: impact of light intensity and exposure time

**Figure 9:**
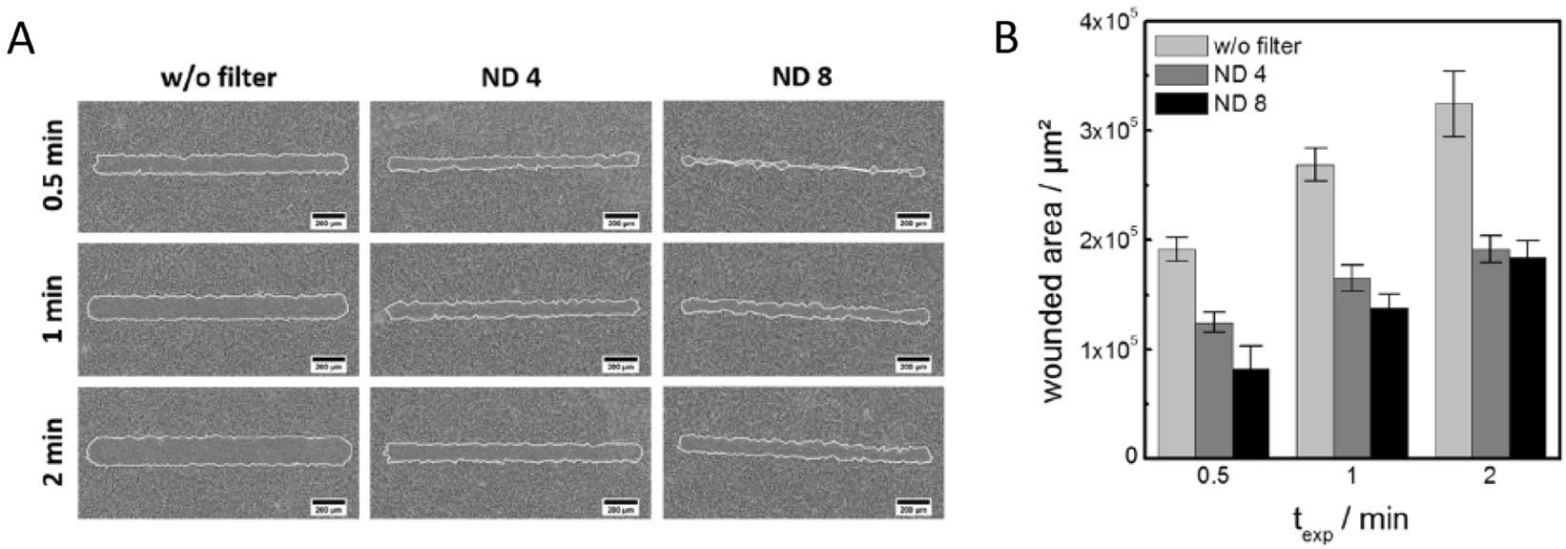
(**A**) Effect of exposure time (t_exp_) and light intensity on wound size when confluent layers of NRK cells were grown on standard photosensitizer-doped functional coatings upon irradiation by a 408 nm laser using an upright CLSM. The exposure time was gradually increased from 0.5 min, 1 min to 2 min. Light intensity was controlled by the use of neutral density filters (ND4 and ND8). Wound edges were delineated by white lines using the ImageJ software. (**B**) Changes in the size of the wounded areas in dependence on exposure time and light intensity. Mean ± SEM; n = 8.

With a series of wounding assays, we addressed the impact of exposure time and light intensity on the degree of cell killing. It is reasonable to assume that either of the two parameters has an impact on the amount of ^1^O_2_ being produced and delivered to the adherent cells. Figure 9 summarizes these assays documented by phase contrast microscopy (A). In either micrograph, the wound was delineated by a white line and the wound size was quantified (B).

The impact of increasing exposure time is revealed in vertical direction, whereas the horizontal direction shows the impact of neutral density filters that reduce the light intensity to ¼ (ND4) or 1/8 (ND8) independent of the wavelength. The optical impression from the micrographs corresponds to the quantitative analysis summarized in Fig. 9B. The longer the exposure time, the bigger is the wound even though the laser scribing pattern and the scan speed were not altered. This result indicates that the harmful impact radiates outwards from the center of the wound. But the increasing wound size is not explained by the ^1^O_2_ diffusion as its life-time is way too short (several µs in water). To our understanding, the intensity profile along the laser path looks like a Gaussian profile. Thus, the intensity is highest in the center of the laser path and it falls off towards the edges. This profile moves back and forth in the direction of the laser many times until the end of the exposure time. The longer the laser is applied, the higher is the cumulated intensity at the edges so that ^1^O_2_ is increasingly produced even at the outer limits of the laser path and the limiting site, where the threshold for cell wounding is surpassed, moves outwards. A similar explanation applies to the increasing wound size with increasing laser power (ND8 → ND4 → no filter). In order to get an estimate of the power density that is applied for cell killing, we have measured the power in the focal plane using a *ThorLab* optical power meter PM500. Since the laser power is affected by the optical components in the illumination path (e.g. illumination pinhole, dichroic mirror), the nominal laser output power is just a rough indicator for this. Using the same settings as in the wounding experiments, we measured a power of 670 µW for the 408 nm laser with no neutral density filter applied. Using ND 4 provided a laser power of 194 µW, ND 8 yielded 92 µW in the focal plane. The damage area was used to normalize the measured power by the area. Accordingly, the power density in the focal plane is 1.9 W/cm^2^ when no ND was applied. For ND4 and ND8 the power density in the focal plane amounts 0.55 W/cm^2^ or 0.26 W/cm^2^, respectively. These values might be useful for setting up the assay on other microscopes with different light sources to reproduce our results.

Similar observations regarding the dependence of wound size on illumination time were made when wide-field fluorescence microscopy was used for excitation (cf. Figure S3). The wound size was about 5% bigger in area than expected from the diameter of the field diaphragm for short exposure times (1 min) but this difference increased with increasing time of exciting the photosensitizer. These phenomena have to be considered when planning on a certain wound size. However, since the impact of exposure time and intensity are well-defined and reproducible, the wound size is precisely and predictably controlled by the wounding parameters.

### Details of the wounding process: how much photosensitizer is needed?

Since the wound size turned out to be dependent on light intensity and exposure time, it was straightforward to hypothesize that it will also be dependent on the concentration of the photosensitizer in the polymer matrix. Moreover, the photosensitizer PtTFPP is the most expensive component of the functional layer. Thus, we prepared polystyrene coatings with concentrations of PtTFPP below 3% (w/w) and compared them with our standard recipe. Figure S4 (cf. Supporting Information) shows phase contrast micrographs that have been recorded after optical wounding of confluent NRK cell layers. The cells were grown on functional coatings doped with 3%, 2%, 0.5%, 0.2% or 0.1% (all w/w) of PtTFPP and wounded by three different exposure times. For each exposure time, the wound size depends on the concentration of the photosensitizer in the functional layer. Using a concentration of 0.1% (w/w) did not provide any cell wounding anymore. A concentration of 0.2% was sufficient to introduce a lesion if the substrate was illuminated for at least one minute. This data confirms the dependence of wounding efficiency on the concentration of the photosensitizer. It also shows that it is possible to prepare a functional coating suitable for cell wounding with concentrations of PtTFPP as low as 0.2% (w/w) which results in a corresponding cost reduction. Only those PtTFPP molecules residing within a few nanometers from the surface contribute to cell killing since the diffusion of ^1^O_2_ during its lifetime is rather limited in the polystyrene matrix. Thus, it seems straightforward to reduce cost for the sensitizer by preparing thinner functional layers. However, since the coating process (spin coating) was found to be more reproducible for thicker layers, we relied on coatings with an average thickness of 1.6 µm as detailed above. We are aware that other coating techniques may allow for thinner and more cost-effective functional layers.

### Wound healing experiments under flow conditions

One advantage of optical wounding is that it does not require any mechanical manipulation of the cell layer (e.g. scratching) or removal of any barrier. Wounding is conducted contact-free and it only requires optical access to the cells to be studied. As such, the assay is also applicable to adherent cells cultured in closed vessels like, for instance, microfluidic devices including complex systems like organs-on-a-chip. To demonstrate applicability of the assay to cells typically grown in microfluidic channels under continuous flow conditions, we have grown human umbilical vein endothelial cells (HUVECs) to confluence in a flow channel.

**Figure 10:**
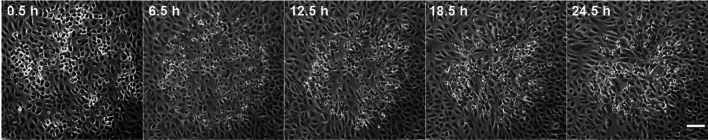
Migration of HUVEC cells under flow after optical wounding in a closed microfluidic channel. The phase contrast images show a time-lapse recording of cell migration on the photosensitizer-doped substrate under continuous unidirectional flow from left to right. The first microphotograph was taken 30 min after wounding. Wound healing progress is documented in 6 h intervals. Scale bar: 100 µm.

The flow channel was customized by coating the bottom with a photosensitizer-doped functional layer in its standard composition. After the cells reached confluence, the unidirectional medium flow was set to 7 dyne/cm^2^ and a circular wound was introduced by means of optical wounding using a wide-field fluorescence microscope. Wound healing under flow conditions was monitored by phase contrast microscopy. Figure 10 summarizes the progress of wound healing starting 0.5 h after wounding in 6 h intervals. The direction of flow is from the left to the right. The micrographs reveal a similar wound healing pattern as observed for non-flow conditions. Time-lapse recording of this experiment is available from the publisher’s website.

## Conclusion

The optical wounding assay as presented here relies on a simple, photosensitizer-doped polystyrene coating of regular cell culture dishes. The final dishes have proven to be equally cell culture compatible as UV-treated polystyrene culture ware and their handling is alike. Due to the homogeneous coating, any spot of the growth surface is addressable for wounding and may be selected flexibly after microscopic inspection of the cell culture, for instance, in co-culture experiments to select a certain subpopulation for migration analysis. Since the generation of ^1^O_2_ is very local and confined to the site of illumination, multiple wounds of variable size and geometry may be introduced into a single cell layer enabling multiple parallel recordings of cell migration from a single well. Due to the ease of and compatibility with large scale production, the new assay may enable high-throughput assays as well. The functional coating is stable and allows for long-term storage in the dark for several months without significant loss of functionality. The assay in its basic form neither requires any additional hardware as it goes well with the light sources integrated in simple fluorescence microscopes nor any different cell culture protocols. It is compatible with protein coatings of the growth surface (data not shown). The wound size is highly reproducible and allows microscopic precision down to the single cell level as well as wounding of large cell areas. Using wide-field microscopes, the shape of the wound is easily controlled by shadow masks introduced into the incident light path. This control of the wounded cell area by the illumination pattern opens up new experimental options to study the impact of wound size, geometry or cell density in the wound. The assay is compatible with closed cell culture vessels like flow channels as long as the channel is made from materials transparent to visible light. This particular feature of contact-free wounding may pave the way to enable wound healing studies even in complex organ-on-a-chip systems.

Regarding the state of the wound, it is important to recognize that the wound contains cell debris which is very different from the popular scratch or barrier assays. Cells migrating in from the periphery of the wound have to remove the cell remnants by proteolysis using extracellular proteases as, for instance, matrix metalloproteinases. This particular aspect of optical wounding provides a wound that resembles the situation in a real wound or during metastatic dissemination from a primary tumor more closely. As such, the assay may allow for a new type of migration assay that mimics more features of the pathophysiological situation to be studied.

### Supplementary Information

Supplementary Information.

## Data Availability

The datasets used and/or analyzed during the current study are available from the corresponding author on reasonable request.
