# Supplementary material for: A high-precision wound healing assay based on photosensitized culture substrates
Source: Sci Rep. 2024 Apr 20;14:9103. doi: 10.1038/s41598-024-59564-9 (PMC11032384; doi:10.1038/s41598-024-59564-9)
Supplement: Supplementary file 1 — Supplementary Information. [file 41598_2024_59564_MOESM1_ESM.docx]

**A High-Precision Wound Healing Assay**

**Based on Photosensitized Culture Substrates**

Saphia Azzam^1,*^, Lea Tomasova^2,*^, Carina Danner^1^, Michael Skiba^1^,
Maren Klein^1^, Zeno Guttenberg^2^, Stefanie Michaelis^3^, Joachim Wegener^1,3,#^

^1^ Institut fuer Analytische Chemie, Chemo- & Biosensorik, Universitaet Regensburg, Universitaetsstr. 31, 93053 Regensburg (G)

^2^ ibidi GmbH, Lochhamer Schlag 11, 82166 Gräfelfing (G)

^3^ Fraunhofer-Institut fuer Elektronische Mikrosysteme und Festkoerper-Technologien EMFT, Universitaetsstr. 31, 93053 Regensburg (G)

* Both authors contributed equally to this study.

# corresponding author

**SUPPORTING INFORMATION**

**Materials & Methods**

*Preparation of Photosensitized Culture Substrates*


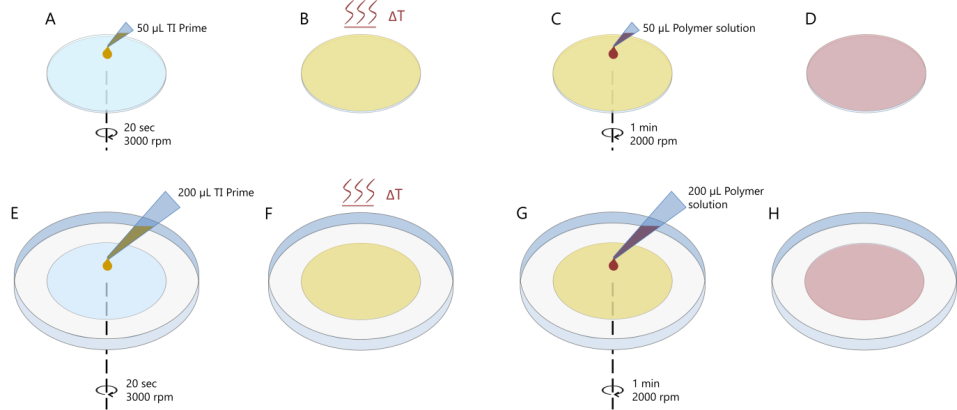


**Figure S1:** Preparation of the functionalized substrates via spin coating. **(A)** The adhesion promotor *TI prime* is deposited on the carrier material by spin-coating for 20 sec at 3000 rpm and activated for 10 min at 130 °C **(B)**. The photosensitizer-doped polymer solution is then spin-coated upon the activated glass surface for 1 min at 2000 rpm **(C)**. After curing in air for one day to ensure solvent evaporation, the functionalized substrates are assay-ready.

**Results & Discussion**

*Characterization of the Photosensitive Coating*


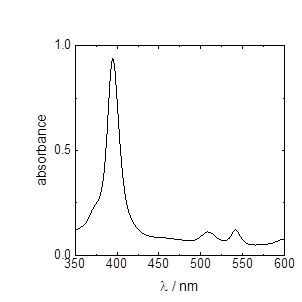


**Figure S2:** Absorbance spectrum of 3 % (w/w) Platinum(II)-5,10,15,20-tetrakis-(2,3,4,5,6-pentafluorophenyl)-porphyrin (PtTFPP) in a polystyrene (PS) matrix. There are three absorption maxima at λ = 395 nm (Soret band), λ = 508 nm and λ = 541 nm. A wavelength of λ = 408 nm was used for optical wounding.

*Details of the Wounding Process: Impact of Light Intensity and Exposure Time*


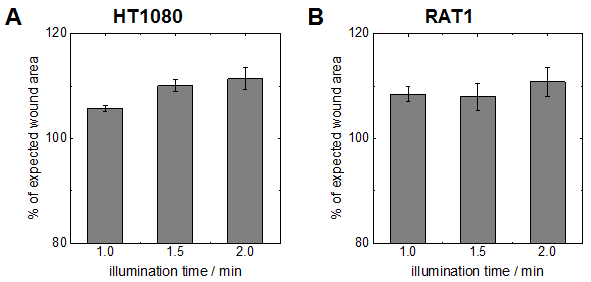


**Figure S3**. Wound areas as quantified from phase contrast micrographs were normalized to the expected wound area as determined from the geometry of the field diaphragm for HT1080 cells (**A**) and RAT1 cells (**B**). The photosensitive layer was prepared according to the standard recipe. The substrates were illuminated at an epifluorescence microscope with a wavelength of λ = 408 nm and a 10x objective for 1 min, 1.5 min or 2 min. The illuminated area was manually controlled by a field diaphragm. Mean ± SD, n=2.

*Details of the Wounding Process: How much Photosensitizer is Needed?*


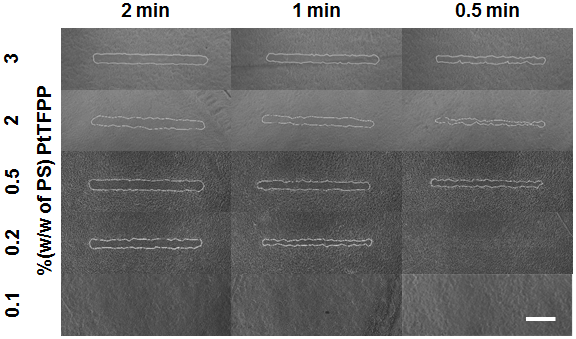


**Figure S4**. Phase contrast micrographs of confluent NRK cells grown on glass substrates that were coated with the photosensitizer-doped functional layer after optical wounding with a laser of λ = 408 nm (CLSM). The substrates were doped with different PtTFPP concentrations between 0.1 %, 0.2 %, 0.5 %, 2 % and 3 % (w/w of PS). The exposure time was set to 2 min, 1 min or 0.5 min, respectively, using an ND4 filter. The wound edges were marked with the help of the image analysis software ImageJ. Scale bar: 400 µm.

*Wound Healing Experiments under Flow Conditions*

A time-lapse video sequence visualizes confluent HUVEC cells grown and optically wounded in a microfluidic channel under flow conditions.

- LINK to PUBLISHER WEBSITE

*Proof-of-Concept Experiments*

**Benchmarking experiments: Optical Wounding versus Barrier Assay (insert)**

Figure S5 displays the effect of 10% fetal bovine serum (FBS) on the wound healing rate of L929 mouse fibroblasts (n=2). Two assays were compared: the optical wounding assay (left) and a standard barrier wound healing assay (right). Both assays correctly identified the stimulatory impact of 10% FBS on wound healing. The optical wounding assay was performed as described in the main manuscript, leaving dead cells in the wounded area. For the barrier assay, the ibidi Culture Insert 2-Well (ibidi, Gräfelfing, Germany) was applied. In this assay, the cells are cultured in two silicon wells that are separated by a 0.5 mm wide barrier, until reaching confluency. By removing the insert, a cell-free wound (gap) is created between the two confluent cell layers. The L929 cells were seeded in the culture-insert placed in a dish with a PS-coated glass substrate at the density of 50.000 cells/cm² and allowed to grow for 24 hours. After the insert was removed, medium with or without 10% FBS was added to the cells and the wound closure was monitored by phase-contrast microscopy, as described above. The L929 cells were purchased from DSMZ (German Collection of Microorganisms and Cell Cultures, Braunschwieg, Germany) and cultured under standard cell culture conditions in RPMI-1640 medium supplemented with 10 % FCS.


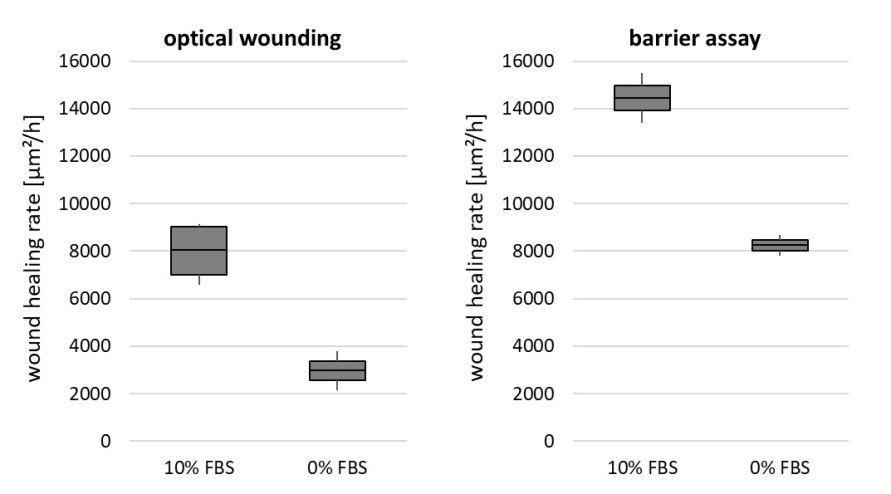


**Figure S5:** Benchmarking experiments comparing the optical wound healing assay to a well-established, commercially available barrier (using ibidi inserts) assay for L929 fibroblasts. In both assays, wound healing was conducted either in serum-containing (10 %) or serum-free medium. Fetal bovine serum (FBS) was used as in routine culture.
